# Factors affecting senior medical students’ career choice

**DOI:** 10.5116/ijme.5c14.de75

**Published:** 2018-12-27

**Authors:** Sophie Querido, Sjoukje van den Broek, Marlies de Rond, Lode Wigersma, Olle ten Cate

**Affiliations:** 1 Central Board for Specialty training for Elderly Care Medicine in the Netherlands (SOON), Utrecht, The Netherlands; 2 University Medical Center Utrecht, Medical School, The Netherlands; 3 Royal Dutch Medical Association, The Netherlands; 4 Dutch Association of Public Health Physicians (VAV), The Netherlands; 5 University Medical Center Utrecht, Center for Research and Development of Education, The Netherlands

**Keywords:** Career preference, specialty choice, medical students, qualitative interview study

## Abstract

**Objectives:**

To gain insight into factors affecting career preference and career choice during the final phase of medical school, above and beyond a model that was presented by Bland and colleagues in 1995 (the "Bland model").

**Methods:**

A qualitative study was conducted. One-hour semi-structured interviews were conducted with final-year medical students about career preference and the factors influencing preference and choice. The interviews were transcribed and a thematic analysis was applied, to identify patterns and interrelationships in the data and to compare and contrast these with the Bland model.

**Results:**

Twenty-four students participated. Three critical sets of factors, not present in the Bland model, emerged from the interviews: (a) factors arising from student-initiated information collection, (b) patient population characteristics of a specialty domain, and (c) the characteristics of teams and colleagues within a specialty.

**Conclusions:**

Students appear to actively match and calibrate perceptions of different specialty characteristics with their current personal needs and expected future needs, and to include cues from self-initiated information collection about a speciality. This agency aligns with Billett's workplace learning theory. Next, specialty patient population features appear to be taken into account; this was not unexpected but not included in the Bland model. Finally, the characteristics of teams and colleagues of a specialty were stressed in the interviews. These three components broaden the applicability of the Bland model--originally created for primary-care careers--to medical specialties in general.

## Introduction

Thinking about medical career choice starts during undergraduate medical education. "What specialist should I become?" is a question all medical students face and must answer.^1^ Most students start medical school with some idea about specialty choice^2^^,^^3^ but first preferences are rarely realized.^3^^-^^5^ At the beginning of medical school, many students have vague images and misconceptions of the medical profession.^6^^-^^8^ Even for those with a strong preference at the start of medical school, career preference is subject to changes during undergraduate training.^6^^-^^8^ Choosing a postgraduate career path is an important choice that is often difficult to reverse once in residency training. Students’ career choices shape the landscape of human resources in health care, and a better understanding of the process of career choice can help to create a better match of students’ preferences with specialty needs.

After medical school, most graduates start residency training. Switching specialty careers during or after postgraduate training is usually difficult and often causes financial and emotional stress for residents.^9^ To change or quit a residency is, therefore, unusual among residents.^10^ This puts pressure on students to choose the right specialty for a lifetime career. The decision, in turn, has a significant impact on career satisfaction and personal well-being later in life.^9^^,^^11^^-^^15^ Specialty choice is not only crucial for the individual but also for society at large, as populations must be served with an adequate mix of medical specialties. Medical students generally determine career preferences without attending to societal needs. Some specialities suffer because they cannot attract enough graduates. A skewed distribution of graduates across the specialties leads to shortages in some specialties and competition in others. Guidance in making the best suitable career choice should, therefore, be part of the medical education, and from this point of view, career preference dynamics are a relevant object of study in medical education.

### Bland model of career choice

Career choice is a process with many interacting factors. A literature review published in 1995 by Bland and colleagues, and recently updated,^16^ features among the most comprehensive summaries of this process to date for medical career choice.^17^ It shows how the process of career choice is essentially a balance between expected future career needs and the perception of the characteristics of a specialty. Career needs are determined by the preferences and values of a student, affected by student characteristics and educational program characteristics. The stronger the perceived similarity between career needs and the perceived characteristics of a specialty, the stronger the desire for this specialty as a career choice.^17^ These career needs change during medical school under the influence of personal development, curriculum, life experience, and specialty experience.^16^^,^^17^ Although this model provides valuable information about the relationship between factors affecting decision and preference of specialty, it was designed to address shortages and geographic maldistribution, which were predicted in the wake of a decrease in the number of medical school graduates entering primary care in the United States in the early 1990s. Secondly, the model was proposed more than two decades ago, and its current applicability is unclear. Nevertheless, the model still provides a valuable lens for investigating factors that influence career choice.

### Aim and research question

Most career preference and choice studies in medicine are specialty specific.^18^^-^^21^ Moreover, many take a quantitative approach^22^ and lack deep insight into students' perspectives on career choice.

This study aims to provide insight into the factors that influence career choice specifically during the final phase of medical school. To reach a deeper understanding of the participants’ views, a qualitative approach was chosen.^23^^,^^24^ The research question we attempted to answer is: Which factors affect career choice of medical students in their final study year?

## Method

### Study design

An exploratory qualitative study was conducted using thematic analysis. Qualitative methodology was used since this study focuses on the experiences and perceptions of medical students; highlighting how this is affected by multiple factors and therefore how their career preferences might be influenced. We conducted 24 individual in-depth interviews to explore senior medical students’ opinions about their career preferences and to relate this to the Bland model.^25^^,^^26^

### Setting

The Dutch educational model allows for variation in study timelines, and graduations in medical schools occur several times per year. During the final-year rotations, students assume increased levels of responsibility in patient care, as they are expected to participate as a junior doctor under strict supervision. In this last year of undergraduate medical training, the medical student in most Dutch medical schools is called a ‘semi-physician’, somewhat similar to a foundation doctor in the UK system or a sub-intern or intern in the USA.^27^ However, successful completion of this year does not guarantee a residency position in a preferred specialty. Dutch graduates need to apply for a residency in an open job market.

### Participants

The study was conducted at the University Medical Center (UMC) Utrecht in the Netherlands with two groups of final-year medical students. The duration of UMC Utrecht’s undergraduate medical curriculum is six years, and the final year is created as a transitional year towards residency and contains predominantly elective clinical and science rotations.^28^^,^^29^ Each sub-cohort starts with a six-week non-clinical block, with the starting dates for these blocks spread out over the final year. The study was explained during lectures in the first week of two such sub-cohorts, once in May and once in October. The lectures were attended by a total of 69 students and all these students were sent a follow-up information email and were offered the opportunity to sign up for the study. After one week, a reminder was sent to all non-responders. This convenience sample included all students starting in these two modules. (24) The study was approved by the Netherlands Association for Medical Education Ethical Review Board (ERB number 308). The participants were informed that participation was voluntary, that confidentiality was secured and that non-participation would not be held against them. They could withdraw from the study at any time without giving a reason. Written informed consent was obtained from all the participants. To preserve anonymity, every participant was asked to choose a pseudonym that was used for data storage and analysis.

### Data collection

We conducted semi-structured, in-depth interviews stimulating respondents to talk freely about sensitive matters. The interviews were held in a quiet studio at UMC Utrecht and lasted one hour. The interviews were planned following participants' availability options. The first interviews were conducted by two researchers (SQ and SB), to encourage similar interview styles in subsequent ones. Furthermore, the interviews were equally divided among the two researchers and were performed face to face. All interviews were audio recorded and during the interview field notes were made for reference during the interview. To further increase the study’s credibility, member validation was performed by writing summaries of each interview and sharing them with the participants.^24^ This did not lead to essential changes; three students made minor changes at the sentence and linguistic level. The topic-list with questions that guided the interviews was based on what was known from the literature on career choice. The list was piloted with six medical students and was refined prior to the interviews with the sample group subjects. The items focused on the participants’ career preference and included questions such as, what are your career preferences? Can you explain these? How familiar are you with these specialties? Which are your electives during the transitional year and why did you choose these? Follow-up questions were used to probe explanations of the answers more deeply.

### Data analysis

The researchers (SQ and SB) had several meetings to discuss the developing analysis. This promoted alignment of the researchers’ individual interpretations and enhanced reflection.^26^^,^^30^^,^^31^ The interviews were transcribed verbatim using a transcription company with participants’ personal data being de-identified. All transcripts were checked against audio recordings by the first (SQ) or second (SB) author.

Both authors read each transcript twice to familiarize themselves with the contents. The authors analysed all data using the lens of the Bland model. The known "student factors" (as opposed to factors that are determined by the educational institution, i.e., status, salary, work-life balance, interesting work, altruism, experiences, role models)^16^ formed the basis for a coding template for describing factors influencing medical career choice.^32^^,^^33^ The quotations were first identified as expressions of a particular factor and further categorised into the sub-themes (e.g., incoming values, career needs to satisfy). The raw data were coded line by line in an open coding process to identify any factor that seemed to influence a student's medical career choice.^32^^,^^33^ Coding and analysis of all interviews were performed by the first researcher (SQ). For analytical rigor, the second researcher (SB) also performed an analysis of four interviews selected to reflect variation in gender, transitional-year start date (May or October), and career preference (intramural or extramural medical specialties). Thus, both researchers were familiar with all different codes and themes.

The coding scheme, as well as the themes, were discussed with the researchers’ team (SQ, SB, MdR, LW, OtC) during several meetings throughout the analysis. This allowed for alignment of the researchers’ individual interpretations and enhanced reflection, to increase credibility in the interpretations of the data.^30^^,^^31^ The Bland model was used as a starting point for analysis to understand factors of influence for medical career choice, both to provide guidance and to be supplemented with new findings during the process. Data analysis continued by importing all data in a qualitative software application (Dedoose).^34^ With the number of interviews, saturation was reached to understand the range of considerations of medical students with respect to their career preferences.

### Reflexivity

The two researchers (SQ, SB) who conducted the interviews were both trained in interview methods. SQ has a Master of Science degree in Health Care Management and was a policy advisor at the Royal Dutch Medical Association. She was not known to the participants and could work primarily as a researcher with an outsider perspective, with knowledge of career options, curricula, and the theoretical framework. SB graduated from medical school at Utrecht University and works as a medical educator and researcher at University Medical Center Utrecht. She was not acquainted with any of the participants but had inside knowledge of the Utrecht medical education program from her own experience. Two co-researchers (MdR and LW) had an outsider perspective, not being involved with University Medical Center Utrecht’s medical school, and one co-author (OtC) had an insider view, being the director at the Center for Research and Development of Education at University Medical Center Utrecht at the time. All three supervising researchers (MdR, LW, OtC) have theoretical knowledge of career choice, to ensure there was consensus for the use of the codebook. This insider/outsider status of the researchers allowed us to understand and interpret the scope of career options as well as the way they emerge in clinical education.

## Results

Senior medical students participating in the study ranged in age from 23 to 26 and included 20 women and 4 men. There were no married or divorced students, and none had children. Sixteen students had a partner, 6 were single, and of 2 the relationship status was unknown.

Major interview themes and sub-themes were identified using the Bland model as a tool for analysis. Three main themes were classified: student characteristics, needs to be satisfied and perception of speciality characteristics. All three themes have a diversity of subthemes. These are listed in Table 1 and further elaborated upon in the subsections below.

### Student characteristics

The students talked about various personal aspects such as where they live, their age, their personality, their relationship status, and so on. With the Bland model in mind, we categorized these under the theme of student characteristics. We identified three student characteristics of note in the interviews: geographic origin, parental education and personality.

**Table 1:** Overview of factors mentioned by interviewees

Theme	Sub-theme
Student characteristics	Geography
	Parental profession
	Personality
Needs to be satisfied	Personal needs
	· career counselling
	· career options
	· lifestyle
	· location/type of practice
	· opinion of others
	· parental preferences
	· status
	· working hours
	· work-life balance
	Societal needs
	· altruism
	Content interest needs
	· characteristics of team and colleagues
	· intellectual satisfaction
	· interesting nature of work
	· patient characteristics
Perception of speciality characteristics	Experience with the specialty
	· experience before study
	· experience in courses & rotations
	· extracurricular experience
	· experience with role model or mentor
	· personal experience
	Information about the specialty
	· representation in the media
	· student-initiated information collection
	Market dynamics of the specialty
	· chances to obtain a residency position

#### Geography

Where to live during and/or after residency appeared of critical relevance for some students. Some wanted to stay in a particular city while others wanted to go back to their family, for example. For some, geographic considerations weighed strongly in making a career choice.

“…That is really important for me. I definitely do not want to leave Amsterdam. I am prepared to travel, but I’d rather not. I probably will have to, but I'd rather not travel more than one hour per day.” (no 18, female)

#### Parental profession

Some interviewees explained how parents' or close family-members' medical professions affected their career choice.

 “…I think that my preference indirectly has something to do with my father’s specialty, but I cannot really explain how. At first, I just did not want the same, but it's in my mind all the time, and I think that played a role. Now I also want to be a gynaecologist, just like my father.” (no 16, female)

#### Personality

Some students elaborated on why their personality would fit with their career choice.

“…I am really interested in people. I also think that is important for an intellectual disability physician or general practitioner [which I consider as career options]. I am always very interested in my friends, how they really are feeling. I think it is important to be sincerely interested and not pretend. I think that my communication skills fit well with these two specialties.” (no. 13, female)

### Needs to be satisfied

All medical students have needs that they wish to fulfil in their future careers and their professional lives and these differ from person to person. These needs may be categorized in three: (1) personal: features that match personal desires around lifestyle, location/type of practice, working hours and work-life balance. Also included here are career features that are important to students because they line up with preferences of other people important to students, sub-themes are career counselling, career options, opinion of others and parental preferences; (2) features that match broader societal needs, the students mentioned altruism and (3) features that align with the content and habits of the specialty, such characteristics of team and colleagues, intellectual satisfaction, interesting work and patient characteristics. Some examples are described below.

#### Personal needs

##### Work-life balance

Many students (both men and women) mentioned the need for a work-life balance.

“… the work-life balance weighs for me. If you want children and a wife then part-time work is attractive. I would like to work hard, but for me it is important that at some time the working day ends. I want to finish at the end of the day and go home and not be called in again. That consideration influences my career preference at the moment.” (no 17, male)

There are also students who notice a work-life imbalance but are willing to accept this because of others factors that are more important such as the content of the work they expect to be doing. An example is:

“… I think about cardiology. It is sort of the only preference I have, although I also think of general practice as an option. Basically, I would like to focus on cardiology. … I think working hours will be heavy, but I do not think that is a reason to lose interest. It will be of big impact on my life, but I am willing to sacrifice that for it.” (no. 24, female)

##### Career options

Most interviewees weighed advantages and disadvantages of preferences to continue searching for the best fit with a specialty and shared these considerations with family, partners, friends or student counsellors.

“…Okay this is the situation, I think I will fit in. Well hhhmm... It is just a process which I can share with my family and best friends and talk about it… about my considerations and so…I am a person of self-reflection and hhhmm... I contrast pros and cons and figure things out for myself.” (no.15 male)

##### Opinion of others

Some students expressed influences by parents or friends (sub-theme "opinion of others"):

“My boyfriend, well yeah…, he is not so happy with my preference for psychiatry.”

Question: “Why?”

“Because he thinks it will be very heavy. And also, because he thinks it is not so cool. He literally said that.”

Question: “What does his opinion mean for you?”

“Yes, hhhmm, on the one hand it makes it difficult for me, but on the other hand not so much, I think I love psychiatry. You know, I find it difficult when important people around me reject it.” (no.5, female)

“...My mother does not care what I end up choosing. She says:” You started medicine, just finish it.” My father tries to restrain himself, but I know he really would love for me to become a medical specialist; sometimes he is a bit pushy. He does not refer to a particular specialty, but he thinks it is important that I choose one with good job opportunities. So, I feel need to go for a hospital speciality.” (no.14 male)

#### Societal needs

##### Altruism

Some students explain about the wish to help people or to be a difference to people.

“Actually, if I want to combine my social commitment with my job and really want to help, I can choose one of those two preferences.” (no.2, female)

#### Content interest needs

##### Characteristics of team and colleagues

The characteristics of team and colleagues appeared to be important, i.e. students sought to be part of the team, to participate in the teamwork, to have colleagues and to identify with the features of colleagues.

“…Yes, this specialty really got me, I liked that. I am attracted by a nice team and this was a nice team.” [..] Yes, the entire team, the gynaecologists and the residents together. I did not experience that before, where people respectfully work together and at the same time enjoy work and are not too serious all day. I feel at home with them. […] I do like doing things on my own, but I really want to work in a team, definitely.” (no.6, female)

“…I think it is also the colleagues that I see, they are like me. So, it is the combination of the specialty and the colleagues.” (no.20, female)

##### Patient characteristics

Patient population characteristics appeared also important. This includes frequency of patient contacts, duration of patient contacts, type of relationship with patients and type of patients (e.g., children or elderly).

“…I just like old people. As a student, I worked a year in elderly care and that really brought me pleasure. I enjoy being in contact with those old people." (no.2, female)

### Perception of speciality characteristics

When asked about previous knowledge and experiences interviewees recalled what they had experienced or heard and how this influenced their image of the speciality. The perception of specialty characteristics may be categorized in three: (1) Experience with the specialty, such experience before study, experience in courses & rotations, extracurricular experience, experience with role model or mentor and personal experience; (2) Information about the specialty such representation in the media and student-initiated information collection and (3) Market dynamics of the specialty as chances to obtain a residency position. Some examples are described below.

#### Experience with the specialty

##### Experience in courses and rotations

All students referred to previous experiences during courses and rotations.

“…I took a clerkship in gynaecology in Africa. That went very well. I was in the fourth year and I had already done a gynaecology block and with this clerkship I really found out that I like this. During my first block, here in the hospital, I was not allowed to do much, just watch. In Africa, I was allowed to do more and I think that this confirmed my interest …this could be it for me.” (no.16, female)

 “Without an internship, you don’t know exactly what it contains. Yes, and when I started my rotations, that was the rotation, which I enjoyed most. Yeah, that is, hhhmm that is it.” (no.1, female)

#### Experience with role model or mentor

Clinicians from a desired specialty would sometimes be influential as an acting role model or mentor.

“One of the general practitioners during my internship, she was a role model for me. How she treated and communicated with her patients, her knowledge, everything she did. I really saw an example in her and I want to be like her when I become a general practitioner myself.” (no.14, male)

#### Personal experience

Some students had experienced health care as a patient or had witnessed a close family member to be a patient.

“…my niece is mentally retarded. She also lives in a special hospital. She enjoys my visits, as do the other patients of her group... And I really enjoy it myself; it is very nice. That is also how I noticed my interest in this specialty.” (intellectual disability physician) (no.13, female)

#### Information about the specialty

##### Student-initiated information collection

Another factor we labelled "student-initiated information collection", signifying extra-curricular activities medical students proactively undertake to gather information to make a career choice, such as voluntarily shadowing doctors in a preferred specialty, visiting medical career events or workshops, participate in a medical career event committees et cetera. These activities provide them with more information or the possibility to check their personal questions with someone with more knowledge or experience within the specialism. One student seeking to explore her match contacted two gynaecologists to ask questions and experience a working day with them.

“…I shadowed gynaecologists for two days and had multiple conversations with them saying 'this is who I am and these are my plans'. I asked for their opinions and suggestions… I'd much rather hear this from gynaecologists themselves directly. It was really useful to have them "precept" my plans and to receive information about what to do and how to present myself if I were to apply for a residency position.” (no.22, female)

And another student explained:

“Once a year there is a career event of tropical medicine. The stories and the experiences of these doctors, yes, they really got me.” (no.23, male)

#### Market dynamics of the specialty

##### Chances to obtain a residency position

Another factor of how student’s perception of a specialty is and was mentioned by them, is the chance to obtain a residency position. In the Netherlands, some specialities and residency positions are more popular than others and there is a skewed distribution in applications for postgraduate medical education programs and varying entrance criteria. Some programs now require a PhD degree. For the one student, this means to put in more effort and the opportunity to show their motivation, while other students are not prepared to invest in these rare changes and maybe to be disappointed not to reach their career choice. One of the medical students said:

“…It is really hard to obtain a residency position. There are so many rival competitors, you need to have a good cv. I tried to work on that, but I am not sure that is sufficient to get my residency position.” (no.1, female)

## Discussion

Our study focused on the factors influencing career choices of final-year medical students. The results are thematically organized through the lens of the Bland model.^16^ Our study was not meant to validate and weigh factors in the Bland model but to use the model as a lens to interpret and categorize our findings and supplement the model if useful.

Senior medical students mentioned factors under three categories of the model: student characteristics, career needs, and perception of specialty characteristics. Within these categories, three new factors could be added to those in the Bland model: student-initiated information collection, patient population characteristics of a specialty, and characteristics of teams and colleagues.

Student-initiated information collection reflects how activities or information sources not offered in the regular education program influence a medical student’s career choice. To promote the specialties, information events, one-day courses, or other opportunities to interact with and experience a specialty are organized to attract medical students and graduates.^35^^-^^38^ This aligns with Billett’s learning theory. He emphasizes the role of the workplace as an implicit learning environment wherein the learner negotiates participation in work activities - and so the learning process - with those individuals representing the interests of the workplace.^39^ Students can use the affordances offered by the workplace (and beyond it) in various ways. Student-initiated information collection is an explicit result of such agency. These experiences will contribute, even if in small ways, to changes in their ways of knowing and sense of self. Moreover, it is one more piece of evidence for an account of learning for work which acknowledges the independence of individuals acting within the interdependence of the social practice of work. Educational programs may consider how the benefits of such agency can be communicated with those who start clinical training.

‘Patient population characteristics of a specialty’ is not cited as a separate factor of influence in recent literature but it may be subsumed within other factors and therefore sometimes labelled differently (for example as interesting work, work content, contact with patients, or interest in relationships).^22^ Nevertheless, students mentioned patient population characteristics as an important separate factor and, therefore, it should be classified as a separate factor.

Characteristics of teams and colleagues affect career choices. Being part of a team can have a positive influence. Second-year residents in the UK, interviewed about career choice, stated that experiences with one specialty team could profoundly shape their opinion of that entire specialty, and this could contribute to the attractiveness of a specialty.^40^ This aligns with what our medical students reported and seems to indicate that being regard as a colleague and participating in a team affects career choice.

Some of the many factors included in the Bland model were not mentioned by the medical students,^16^ in particular (institutional:) type of school, mission/structure, faculty composition, admission committee, faculty values, curriculum committee, student composition, institutional culture and curriculum, (student-related:) gender, study year, ethnicity, age, self-confidence, marital status, academic performance, (profession-related) salary, psychical/emotional workload, professional autonomy, scope of self-practice, perception of gender differences and perception of status. We attribute the absence to a lack of awareness among students or not relevant for the Dutch situation. For example, students in the Netherlands graduate with less debt than medical students in some other countries; it is possible that this is why no student in this study mentioned financial burden as a factor of any importance in career choice.^22^^,^^41^^,^^42^In the USA and New Zealand, the cost of education is significantly higher, and graduates are perhaps for this reason more likely to choose a medical career that is more highly paid.^41^^,^^42^ Hence, while the financial burden was not a factor of influence in this study, it should not be excluded, as previous studies with other populations attest to their importance in students’ career choice.

To provide support to medical students in making career choices an overview of all factors influencing this career choice is useful. Our study adds three factors to yield a more complete model of factors of influences on career choice of medical students. It provides a better understanding of how students reflect on their career choice. Knowledge of these factors can be useful for the development of tools and strategies for career support by educators and policymakers.

### Limitations

This study has some limitations. First, this study was performed at a single institution, one medical school in the Netherlands. We have to bear in mind that the institutional environment can influence results. The Dutch situation may differ from other countries, by its transitional year and admission process for residency.^28^^,^^43^ Furthermore, the results of the study are predominantly a reflection of female students’ experiences. The proportion of male and female students in Dutch medical schools (about 65% female)^44^^,^^45^ is different from the proportion in our study population (83.3% female) which could result in bias in the data. In addition, participation was voluntary which may have led to a non-representative sample of the student population as a whole. However, we had a representative mix of participants as we look to their age and specialty preferences.

## Conclusions

Medical students acknowledge many factors to be of influence on career choice including some factors not presented in the Bland model of medical career choice. Our study offers educators and school counsellors better insight into factors of influence and contributes to establishing a more accurate and complete model for guidance of medical students in making a career choice. This can contribute to future career satisfaction and personal well-being as well as to a better distribution of graduates across specialties.

### Acknowledgements

The authors like to thank H. Carrie Chen, MD, PhD, Georgetown University, Washington DC, Marieke F. van der Schaaf, PhD, director of the Centre for Research and Development of Education at the Education Centre at University Medical Centre Utrecht and Anneke van Enk, PhD, researcher, Centre for Health Education Scholarship, University of British Colombia, Vancouver, Canada for their suggestions with an earlier version of the manuscript and textual suggestions.

### Conflict of Interest

The authors declare that they have no conflict of interest.
